# Impact of rare diseases in oral health

**DOI:** 10.4317/medoral.20972

**Published:** 2016-07-31

**Authors:** Ana Molina-García, Lizett Castellanos-Cosano, Guillermo Machuca-Portillo, Manuel Posada-de la Paz

**Affiliations:** 1 Associate professor. Master in Special Care in Dentistry. University of Seville; 2 Assistant professor, PhD in Special Care in Dentistry. Department of Stomatology. University of Seville; 3 Professor of Special Care in Dentistry. Department of Stomatology. University of Seville; 4 PhD in Medicine and Surgery. Director of the Rare Diseases Research Health Institute Carlos III. Ministry of Economy and Competitiveness

## Abstract

**Background:**

Rare diseases (RD) are those that present a lower prevalence than 5 cases per 10.000 population. The main objective of this review was to study the effect on oral health in rare diseases, while the secondary objective of the study is theme upgrade.

**Material and Methods:**

Comparative observational case-control studies were analysed and a systematic review was conducted in PubMed. Each rare disease listed on the statistical data record of the Health Portal of the Ministry of Equality, Health and Social Policies Board of Andalusia was associated with “oral health”. The variables studied included dental, oral mucosa and occlusion alterations, oral pathologies (caries, periodontal disease) and other alterations (mouth breathing, parafunctional habits, etc). A bias analysis of the variable caries was conducted.

**Results:**

Six RD were selected through our inclusion and exclusion criteria (hypogammaglobulinemia, Rett syndrome, Marfan syndrome, Prader-Willi syndrome, cystic fibrosis and Cri du chat syndrome) in a total of 8 publications, of which four trials were classified as high risk of bias and one of them as medium risk. There were not trials with low risk of bias.

**Conclusions:**

The main statistically significant differences found by Syndrome compared to a control group were in Hypogammaglobulinemia with a greater tendency to enamel hypoplasia and dry mouth. The Rett syndrome had, as well, a greater tendency to an anterior open bite, ogival palate, bruxism, mouth breathing and tongue thrusting. Prader-Willi syndrome had a tendency of dental erosion, and Cri du chat syndrome showed a higher association to *Tannerella forsythia*.

**Key words:**Rare diseases, oral health.

## Introduction

The European Commission defines rare diseases (RD) as all diseases with a prevalence lower than 5 cases per 10.000 population (1). In Spain, the Epidemiology of Rare Disease Research Network (REpIER), describes a set of characteristics, of which, at least one of them has to be present in the disease to be recognized as rare: chronicity, lack of knowledge of the etiology of the disease, lack of treatment or poor accessibility, a significant disease burden or limitations on quality of life.

In recent years, the classification of diseases has undergone continuous modifications determined by new genetic findings that subdivide diseases, which were previously under the same category (2). Rare diseases can occur at any age and they present a wide range of alterations and symptoms that vary not only from one disease to another, but from patient to patient according to the degree of affectation and evolution. Manifestations, alterations and oral pathologies can be found within this diversity.

The principal objective of this review was to study the affectation of oral health in RD, based on statistical data recorded in the Autonomous Community of Andalusia. The secondary objective is a theme upgrade.

## Material and Methods

The present systematic review focuses on case-control observational comparative studies in humans with rare diseases associated with oral health. Theses RD are registered in the section “statistical data on rare diseases” of the Health Portal of the Department of Equality, Health and Social Policies of the Regional Government of Andalusia. The cases documented in Andalusia on this list are 76 RD. Since this review does not involve humans, it does not require the Ethics Committee approval.

- Inclusion and exclusion criteria

The inclusion criteria were case-control trials published in English in the last 10 years to date (2004-2014). The exclusion criteria were the articles that did not meet the inclusion criteria and from the list above mentioned the Down syndrome was ruled out since it is not considered a rare disease because of the information collected during 1980-2007 by the Spanish Collaborative Study of Congenital Deformities, it shows that approximately 11 children out of 10.000 born had Down Syndrome.

- Search strategy and data collection

The search strategy was developed using a flow chart described on the article wrote by Urrutia *et al.* (3) about PRISMA declaration. We started the research on July 15th 2014 using PubMed database; we typed the name of each RD from the list of the documented cases in Andalusia, except for Down syndrome (75), “AND” “oral health” in the search field. The last search was carried out on December 30th 2014.

As an initial selection, we read the title and the abstract of each article, except for those that were not associated to oral health. The inclusion criteria were applied to these articles, and the ones that met the eligibility criteria were analyzed using the next variables: country of origin, sample size and sample average age on the study group (SG) and control group (CG). On each article the next characteristics of the patients were reviewed and analyzed: 1. Dental alterations (type of alteration within form, color, position, number and structure). 2. Soft tissue alterations (Tongue: alterations of form and size; Lips: pathologies that affect lips; Soft tissues: pathologies that affect mucous). 3. Abnormalities on dental occlusion: type of malocclusion. 4. Oral pathology (Caries: if patients with a RD are more or less prone to caries than patients in the control group; Gingivitis: if patients with a RD are more or less prone to gingivitis than patients in the control group; Periodontal disease: if patients with a RD are more or less prone to periodontal disease than patients in the control group). 5. Others (Parafunctional habits: thumb sucking, lip sucking, tongue thrusting, bruxism, onychophagia, mouth breathing and any oral characteristics that cannot be included in the above sections).

- Bias analysis

Bias analysis was performed with caries variable, since it has been the most studied in the selected articles. The risk of bias was evaluated in different levels: clinical trial (selection of the control group and statistical analysis), and results (the validity of the measure, the number of examiners, and accuracy) using the seven criteria listed below and that were adapted from the PRISMA declaration (4): 1) a control group included; 2) a control group justified; 3) statistical approach justified; 4) validated the measurement used for dental caries (i.e. Decayed, Missing or Filled Surfaces in deciduous dentition [dmfs],Decayed, Missing or Filled Tooth in deciduous dentition [dmft], Decayed, Missing or Filled Surfaces in permanent dentition [DMFS], Decayed, Missing or Filled Tooth in permanent dentition [DMFT]); 5) different highly trained dental examiners; 6) blind dental examiners; 7) assessed the intra and/or inter reliability of the evaluator. The trials that fulfilled the seven criteria were classified as low risk of bias. The trials that fulfilled four or six criteria were classified as medium risk of bias, and the remaining trials were classified as high risk of bias.

- Statistical analysis

The Kappa index of agreement between the two authors (AM and PM) was calculated. Significant statistical values ​​found according to the variables analyzed in each study are shown in  Table 1 marked with an asterisk.

**Table 1:**
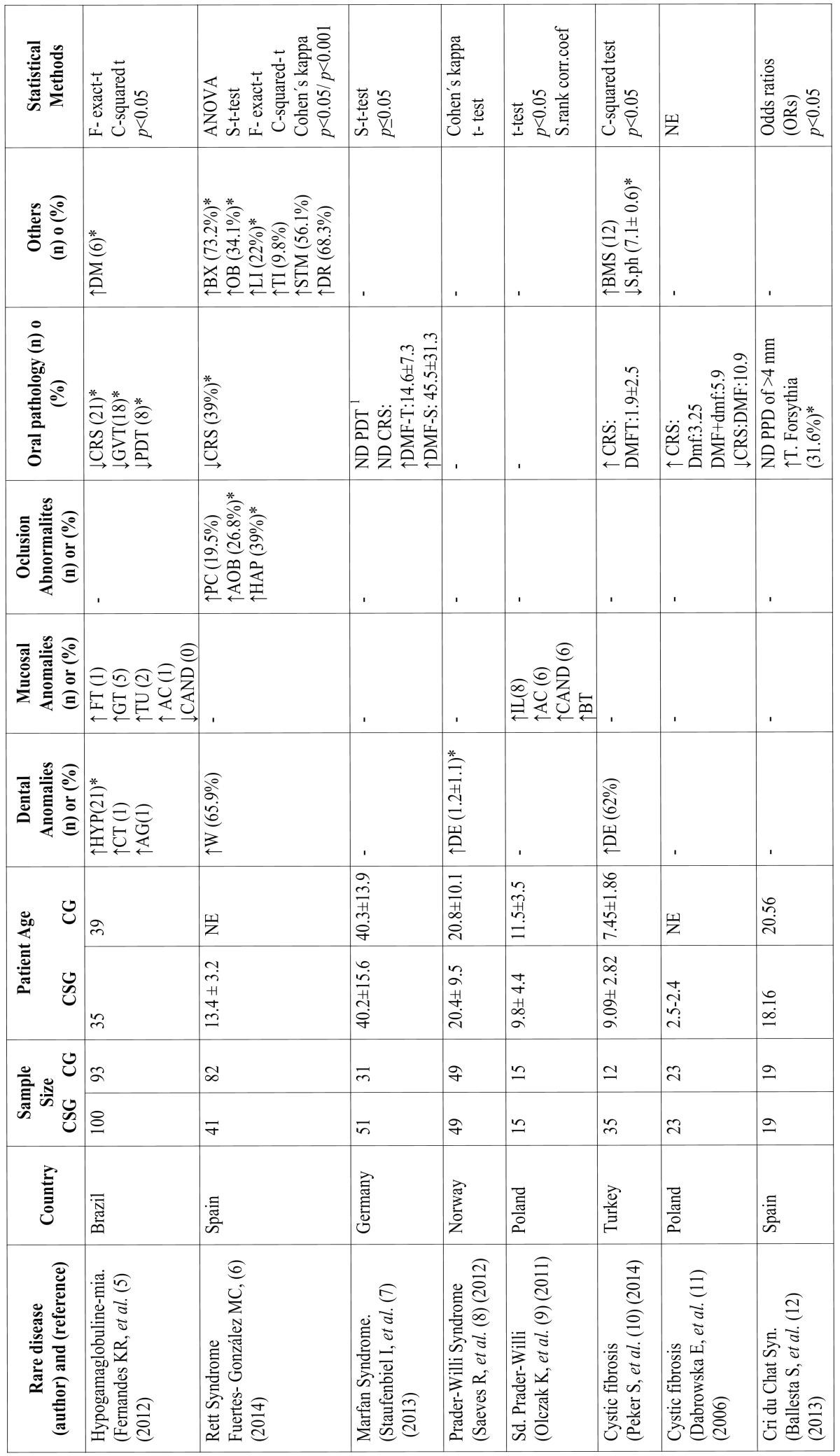
Studied data from the publications obtained.

## Results and Discussion

The main objective of this systematic review was to assess the effect on oral health in rare diseases (RD) we have registered with statistical data in the System Health of the Government of Andalusia.

**Figure 1:**
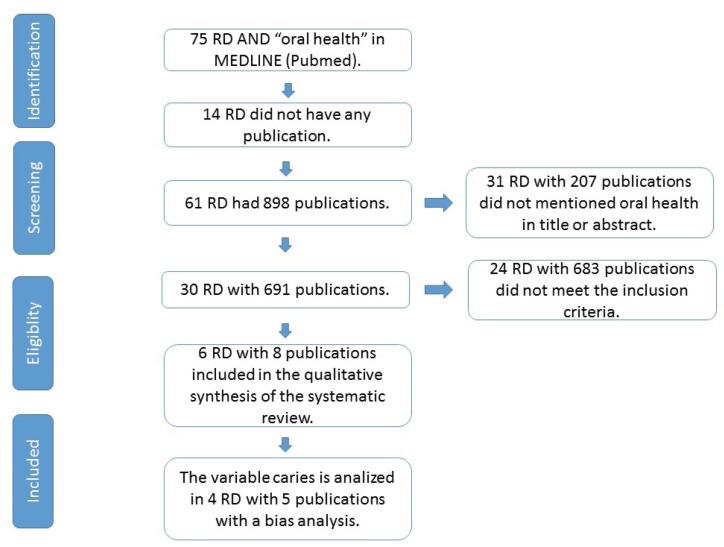
PRISMA® flowchart describing the search strategy and inclusion of the studied articles.

The search performed by the authors (AM and PM) showed 898 publications from the Medline (PubMed) data base. Based on information provided in the title and the abstract of the study, 31 RD were excluded, with a total of 207 publications (Fig. 1). Eight studies met the inclusion criteria (5-12) and were incorporated to the systematic review, the bias analysis of the viable caries was carried out in a total of 5 publications (5-7,10,11) with 4 RD, excluding publications of Cri du Chat syn. and Prader-Willi syn. where this variable was not studied.

The wording of the article was conducted by (AM and LC) and the investigation led by (GM). The Kappa index between both authors (AM and PM) was k > 0.79, obtaining an acceptable agreement.

The data acquired from each publication are shown in  Table 1 (we only specify data about the study group variable and if it increases or reduces in relation to the control group). Most of the clinical trials were published between 2012 and 2014 (5-8,10,12) and the population they include was from European countries (6-9,11,12). The sample size varies depending on the clinical trial, the one that shows the largest sample size is a publication made in Brazil (5) with 100 patients in the study group and 93 in the control group. The average age of all publications ranges between 9 and 21 years old; only two clinical trials (5,7) have an average age between 35 and 40 years old. The variable, oral pathology, is the most studied in the majority of the clinical trials (5-7,10,11) followed by dental alterations. The rare diseases that have the most studied variables are Hypogammaglobulinemia and Rett syndrome. The Chi-squared test (5,6,10) was the most used statistical treatment along with the Ficher exact test (5,6); there is only one clinical trial (11) that did not use any statistical analysis.

Analysis based on risk of bias: Four clinical trials were classified as high risk of bias and one of them as medium risk of bias ( Table 2). There were not trials with low risk of bias. All the clinical trials had a control group, but none of them had a particular reason why this group was selected as control group. Three studies specify the statistical analysis performed. All clinical trials adopted standard measures of dental caries (i.e. dmt and DMF). Most of the clinical trials had only one examiner, and only one of them provided enough information about the intra or inter reliability of the evaluators.

**Table 2:**
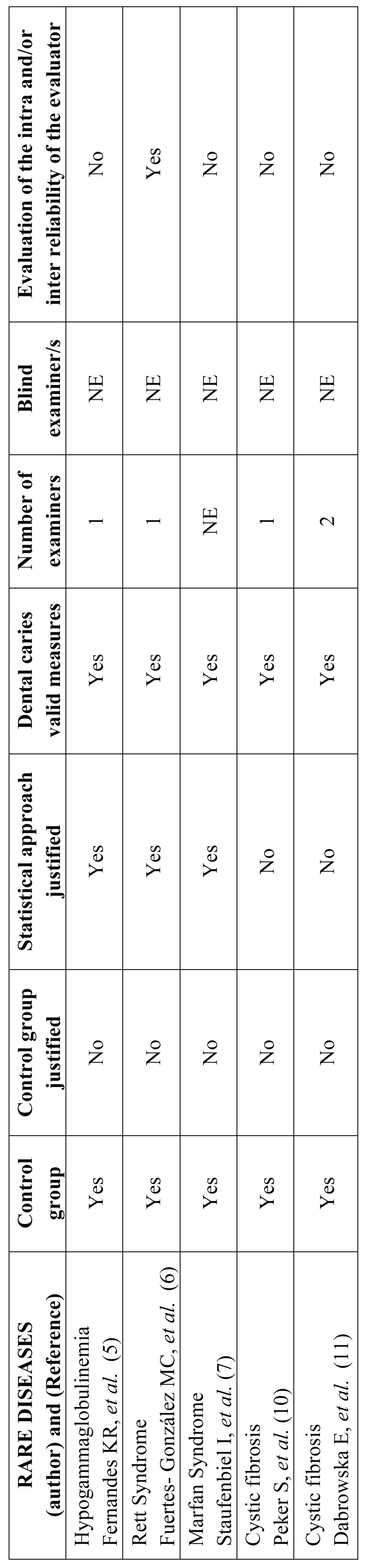
Caries risk analysis.

Nowadays, one of dental research priorities should be to improve the knowledge regarding the relationships between rare diseases and oral cavity, finding new treatment modalities, adapting existing ones and looking for reasonable patient comfort without overtreating. Collaborate with screening programs and treatment of rare diseases must be a dentistry goal. The clinical impact of this systematic review in daily dental practice indicates the need to incorporate systematic prevention and monitoring of these patients in order to decrease the oral pathology associated with rare diseases. In recent decades it has seen a greater social interest in the patients included in this group of rare diseases. One of the current metric of social progress, is the increase in the quality of provided care. Improving the quality of life and social well-being is of vital importance in the community impact. The bibliometric impact that this article can have is relevant as is concerned a social aspect that has not previously been discussed in any study. It is the first systematic review carried out in relation to oral health status in patients with rare diseases.

One of the limitations of our review, was the need for synthesis of such a vast subject as are the RD. We have excluded RD with characteristics that affect oral health that not meet the inclusion criteria, which were case-control studies over the last ten years up to this date (Fig. 1). For this reason, this review suggests that more clinical trials should be carried out to evaluate each RD individually.

The secondary objective of the review is to update the theme. A brief description of general and oral characteristics that can be found in each of the syndromes included in this review are mentioned.

-Hypogammaglobulinemia 

Hypogammaglobulinemia is a group of disorders characterized by low levels of serum immunoglobulins (13). Until January 2013, there were 204 cases recorded in Andalusia with an average age of 62 years, it is high if we compare these numbers with the study carried out in the Brazilian population (5) with a mean value of 35 years of age.

A justified reason was not found about the large difference of age, although it is known that the old-age population ratio in Spain is larger. Regarding the oral characteristics of this disease, no other study was found in the literature in order to discuss the matter.

Hypogammaglobulinemia (5) had dental and soft tissue alterations, oral pathology and “others”. Enamel hypoplasia and dry mouth sensation were statistically significant, in relation to the control group, followed by periodontal disease and caries.

-Rett Syndrome

Rett syndrome is a neurological disorder characterized by the regression of the psychomotor development with autism, head growth deceleration, seizures and a repetitive stereotyped hand movement (14). Fuertes-González *et al.* (15), carried out a literature review between 1985 and 2007 about oral health in people with Rett syndrome and it was observed that only two studies, out of seven, had more than one case with this characteristics; bruxism was the most seen variable, which is in line with the study performed by Fuertes-González *et al.* (6) in 2014 found that of the 41 cases observed among the population of Valencia and Murcia, 73.2% had bruxism, being statistically significant. Rett syndrome (6) had dental and occlusion alterations, oral pathology and “others”. However, anterior open bite, ogival palate, bruxism, mouth breathing and lip interposition were statistically significant. Caries was less significant in the study group regarding the control group.

-Marfan syndrome

Marfan syndrome is an autosomal dominant genetic disease that affects connective tissue elastics fibers, appearing in systems or organs, such as the cardiovascular, skeletal, eye, skin, and lung (16,17). Only one study analyzed the variables caries and periodontal disease, not been statistical significant compared to the control group. (7). However, Morales-Chávez *et al.* (18) conducted a small literature review and found studies as the one from Bauss *et al.* (19) that showed a greater prevalence of pulp obliteration and pulp stones formation. In a clinical trial with 21 cases of Marfan syndrome (20) a high prevalence of the temporomandibular joint disorders were found, they were caused by subluxations and an anterior disk displacement.

-Prader-Willi syndrome 

Prader-Willi syndrome is a complex disorder characterized by hypotonia, severe feeding problems, hyperphagia and obesity during early childhood, low height, facial dysmorphic disorder, hypogonadism and learning and behavioral problems (21). Prader-Willi syndrome had dental and soft tissue alterations (9). Dental erosion was also the most seen variable in the study performed by Saeves *et al.* (8) included in our review where 45 cases with RD had a mean value of dental erosion of 1.15±1.1 regarding the control group [45] with 0.18±0.3. Balleui-Forestier *et al.* (22) carried out their own study with 15 patients with Prader-Willi syndrome and the most seen characteristic was dental erosion, not for the number of cases that had this alteration, because there were only 4 cases, but for the large quantity of dental erosion.

-Cystic fibrosis 

Cystic fibrosis is the most common autosomic recessive lethal disease in Caucasian population, it is catheterized by abnormal concentrations of electrolyte in exocrine secretion and the inability to eliminate mucous secretions, which causes obstruction and accumulation of bacteria in the digestive and respiratory system as well as the reproductive tract. It also causes abnormal levels of chloride in sweat (23). Children and teenagers with cystic fibrosis have a greater risk of dental caries because of four factors related to this disease (24): oral *Streptococcus Mutans* increase 20 times more, gastroesophageal reflux, enamel alterations and a high-calorie diet to maintain weight.

Despite these risk factors, most of the clinical trials of the last 20 years show that people with cystic fibrosis have a low risk of dental caries (25,26). Nevertheless, in 2013 Chi *et al.* (27) carried out a systematic review about caries prevalence in children and teenagers with cystic fibrosis and observed that teenagers that did not have this disease had less caries than teenagers with cystic fibrosis. This result matches with the studies in our review, except for the group between 13 to 24 years old with permanent dentition since they had less caries than the control group (11).

There is a theory that establishes that antibiotics reduce intraoral levels of *Streptococcus mutans*, which in turn reduce risk of caries. This theory may be true if we apply this to children but not in teenagers with cystic fibrosis because when the child is 11 years old, there is a microbiological change at respiratory level that makes *Pseudomonas Aeruginosa* (gram-negative) the predominant bacteria. New antibiotics were developed to solve this problem including inhaled tobramicyn, which is an aminoglycoside that fights against *P. aeruginosa* and it is commonly prescribed for teenagers with cystic fibrosis (28). Since tobramicyn does not affect *S. mutans* bacteria, teenagers with this disease can lose protection against caries. In fact, taking this gram-negative antibiotic inhaled may make an ecologic pressure that favors gram-positive bacteria as *S. mutans*, which elevates risk of caries.

The clinical trials included in this review do not describe enamel alterations, however, different studies (25,26) agree that patients diagnosed with cystic fibrosis have a higher percentage of enamel alterations in permanent dentition, from which the most common is hypoplasia.

Azevedo *et al.* (29) mentioned that probably the high incidence of enamel defects may be a result of a nutritional and metabolic imbalance, as well as, long-term pharmacological treatments, which are often seen in patients with chronic cystic fibrosis.

Regarding dental erosion, it was not statically significant in children with cystic fibrosis or the control group with healthy patients (10). No other study was found that considers dental erosion, however, the esophageal reflux and the inhaled medication that patients with cystic fibrosis take, may have an influence in the presence of dental erosion.

Cystic fibrosis (10,11) had dental alterations, oral pathology and others. It did not have any significant characteristic regarding the control group.

-Cri du chat syndrome 

Cri du chat syndrome was diagnosed, for the first time, as a chromosomopathy because of the partial or total loss of the short arm of 5 chromosome, in 1963 by Lejeune *et al.* (30). Its clinical characteristics include a typical cat-like cry, facial dimorphism, microcephaly, and severe retardation in mental and psychomotor development. Despite being considered as a rare genetic disease, is one of the most common syndromes caused by chromosomal deletion in humans (1:15.000-1:50.000). There are 83 recorded cases of Cri du chat syndrome in Andalusia up to 2013.

Cri du chat syndrome had only oral pathology and was found a statistically significant greater association with *Tannerella forsythia* in the study group (12). This case-control study shows that there may be a microbial predisposition, which causes periodontitis. Some statistically significant differences were observed only regarding *Tannerella forsythia*, because the quantity of this bacteria was higher in the group with Cri Du Chat syn. than the control group. Therefore, the authors conclude that *T. forsythia* is closely associated with patients that present Cri du chat syn., rather than patients in the control group. Besides, other authors used the Community Periodontal Index of Treatment Needs (CPITN) to demonstrate that a small percentage (22.9%) did not show any sign of periodontal disease (31). However, a greater predisposition to periodontal disease may be associated with poor oral hygiene commonly observed in people with mental disability (32).

One study showed that 56.06% of a total of 31 patients with Cri du chat syn. brushed their teeth by themselves without adult supervision (31). The author of this study highlighted the importance of Cri du chat syndrome and oral hygiene, since multiple factors did not help to clarify the association between both, as for example, the diet, the cognitive level of the patient and the way the brushing was performed.

There are more characteristics, which are quite common in this syndrome, for example, mandibular retrognathism that has a percentage of 90.63% over 32 cases with Cri du chat syn. (31) or a 70% over 10 cases (33), ogival palate has a 55.17% over 29 cases and the anterior open bite had a 63.33% over 30 cases (30).

People with this syndrome can also have defects in the dental tissue, being the most common enamel hypoplasia with 54% over 23 patients (31). Moreover, Rodríquez-Caballero *et al.* (31) observed some dental defects with a lower percentage, like dental opacities (33%), dental agenesis (9%), macrodontia (6%), supernumerary teeth (3%), root resorption (3%) and dental transposition (3%). A comprehensive review centered in oral and craniofacial manifestations of this syndrome has recently been published by the authors of this review (34).

## Conclusions

The main statistically significant differences found by Syndrome compared to a control group were: in Hypogammaglobulinemia enamel hypoplasia and dry mouth; in Rett Syndrome anterior open bite, arched palate, bruxism, mouth breathing and lip interposition; in Marfan syndrome no statistically significant differences were found; in Prader-Willi Syndrome dental erosion; in cystic fibrosis no significant differences were found and in Cri Du Chat syndrome increased presence of bacteria *Tannerella forsythia*.
